# Supplementary material for: Unlocking New Avenues: Solid-State Synthesis of Molecularly Imprinted Polymers
Source: Int J Mol Sci. 2024 May 18;25(10):5504. doi: 10.3390/ijms25105504 (PMC11122393; doi:10.3390/ijms25105504)
Supplement: Supplementary file 1 [file ijms-25-05504-s001.zip › ijms-2994243-supplementary.pdf]

Electronic Supplementary Material (ESI)

Unlocking New Avenues: Solid-State Synthesis of Molecularly Imprinted Polymers

Bogdan-Cezar Iacob, Andreea-Elena Bodoki, Diogo Filipe Da Costa Carvalho, Antonio Augusto Serpa Paulino, Lucian Barbu-Tudoran, and Ede Bodoki*

Experimental Procedures

Materials

Analytical grade standard propranolol (PRNL, 99%), carvedilol (CVDL, 98%), alprenolol (ALPRNL) and atenolol (ATNL, 98%) were purchased from Sigma (Steinheim, Germany). Methacrylic acid (MAA) 99%, methylene bis-acrylamide (MBA), ethylene glycol dimethacrylate 98% (EGDMA) and 4,4'-azobis(4-cyanovaleric acid) (ACVA) were purchased from Aldrich (Steinheim, Germany). HPLC grade acetonitrile (ACN) and methanol (MeOH), ammonium persulfate (APS, 98%), and alumina powder (mean particle size 45 μm, purity 99.9%) were provided from Sigma-Aldrich (Steinheim, Germany). Glacial acetic acid was obtained from Merck (Darmstadt, Germany). 2-(trifluoromethyl)acrylic acid (2-TFMAA) was purchased from Apollo Scientific (Cheshire, UK).

Table S1. Composition of synthesized MIPs and NIPs using the conventional and mechanochemical approaches.

#	Synthesis type	Molar ratio			Others	Solvent (μL/mL)
		Template	Functional monomer	Crosslinker/initiator		
		1	6	24		
MIP_M1	LAG[a]	ATNL	MAA	MBA/APS	alumina	ACN (50 μL)
MIP_M2	LAG	ATNL	2-TFMAA	MBA/APS	alumina	ACN (50 μL)
NIP_M1	LAG	-	MAA	MBA/APS	alumina	ACN (50 μL)
NIP_M2	LAG	-	2-TFMAA	MBA/APS	alumina	ACN (50 μL)
MIP_S1	CMI[b]	ATNL	MAA	EGDMA/ACVA	-	ACN (5 mL)
MIP_S2	CMI	ATNL	2-TFMAA	EGDMA/ACVA	-	ACN (5 mL)
NIP_S1	CMI	-	MAA	EGDMA/ACVA	-	ACN (5 mL)
NIP_S2	CMI	-	2-TFMAA	EGDMA/ACVA	-	ACN (5 mL)

[a] LAG – Liquid-assisted grinding. [b] CMI - conventional molecular imprinting in porogenic solvent.

The highest yields of 33% and 25% were for MIP_M2 and MIP_M1, respectively. The corresponding non-imprinted polymers (NIPs) were achieved with 7% and 16% yields. Under the current experimental conditions, these yields are only around half of the values recorded for the solution-based synthesis of imprinted and non-imprinted polymers (55-80%). Nevertheless, higher yields of the solid state synthesis are to be expected using dedicated instrumentation (planetary or vibration ball mills, etc.) commonly employed in mechanochemistry, due to the combination of higher impact energies and higher surface areas associated with the larger milling balls, even in the absence of UV iradiation or the admix of mecanoactivated catalytic surfaces, such as alumina.

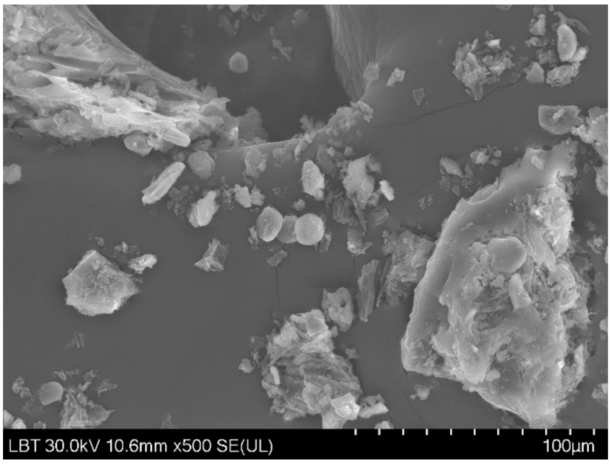
LBT 30.0kV 10.6mm x500 SE(UL)
100µm

A.

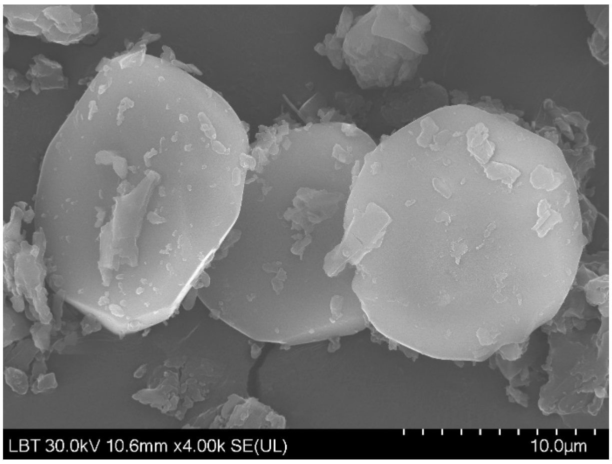
LBT 30.0kV 10.6mm x4.00k SE(UL)
10.0µm

B.

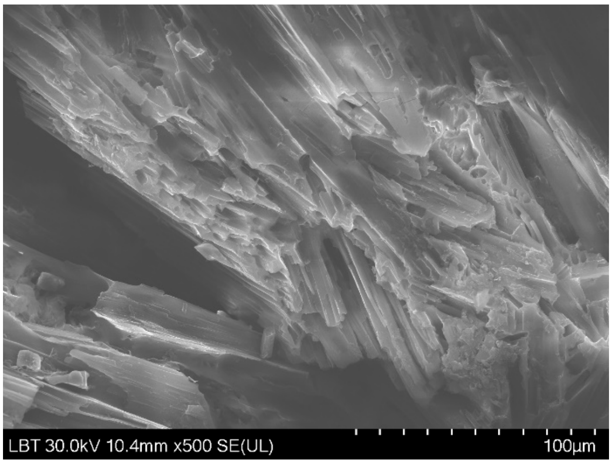
LBT 30.0kV 10.4mm x500 SE(UL)
100µm

C.

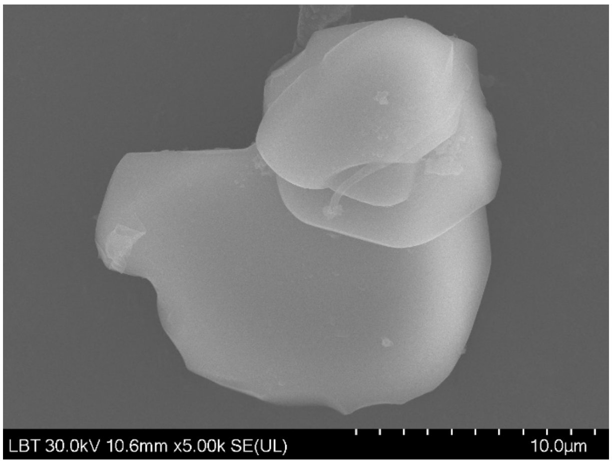
LBT 30.0kV 10.6mm x5.00k SE(UL)
10.0µm

D.

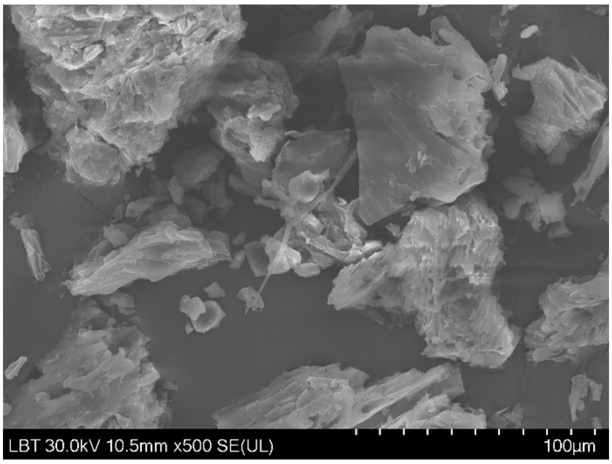
LBT 30.0kV 10.5mm x500 SE(UL)
100µm

E.

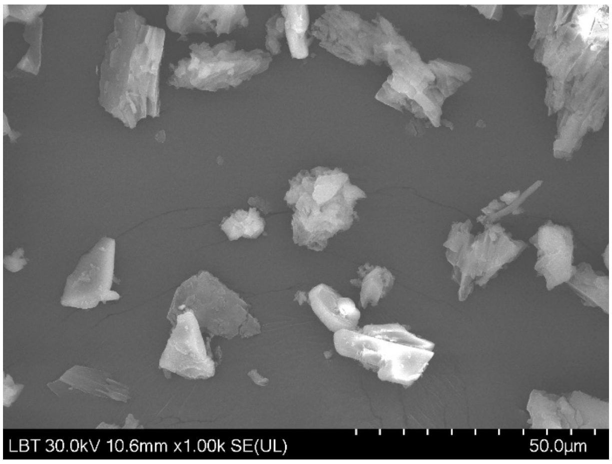
LBT 30.0kV 10.6mm x1.00k SE(UL)
50.0µm

F.

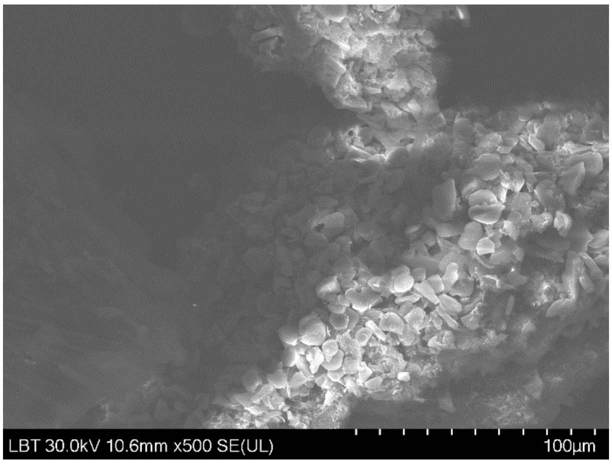
LBT 30.0kV 10.6mm x500 SE(UL)
100µm

G.

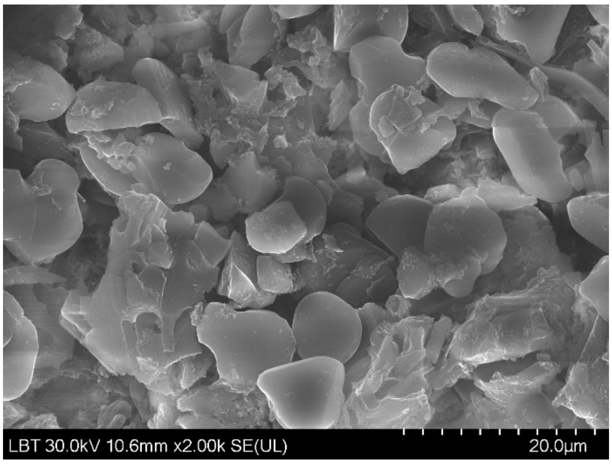
LBT 30.0kV 10.6mm x2.00k SE(UL)
20.0µm

H.

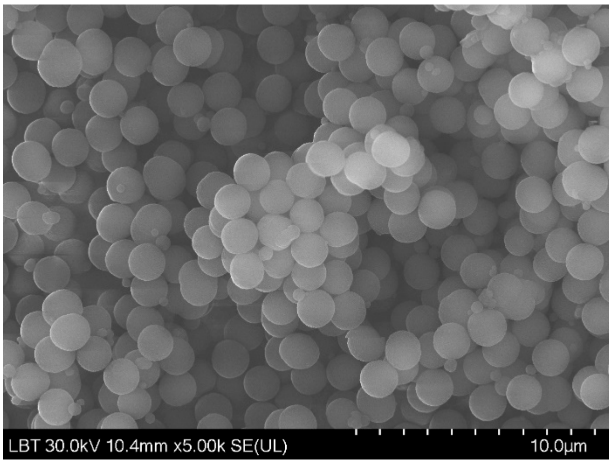

I.

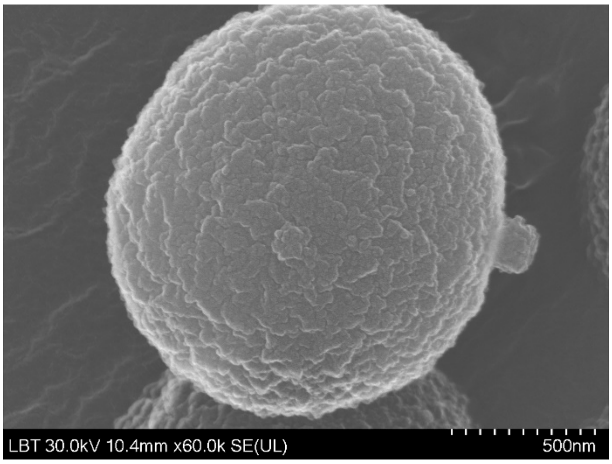

J.

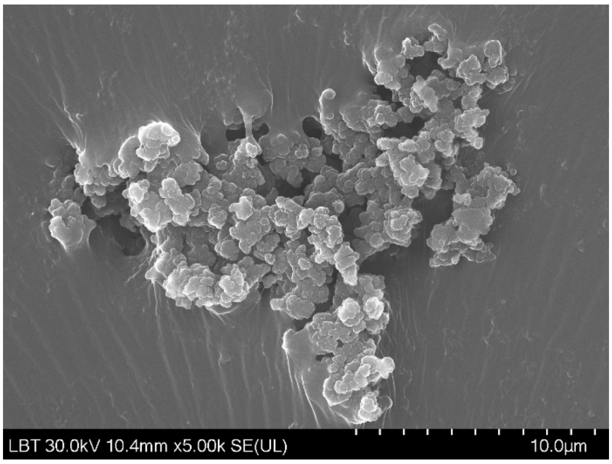

K.

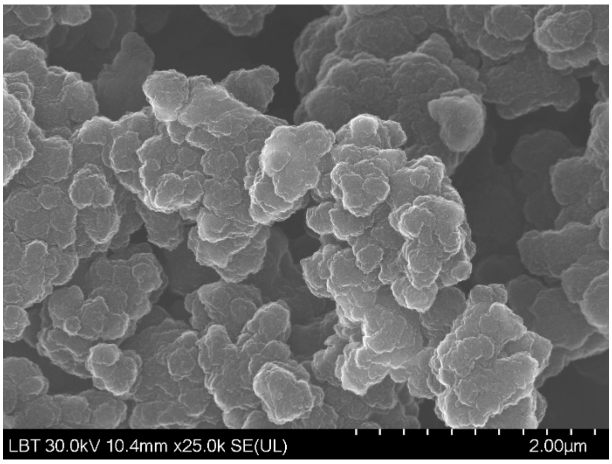

L.

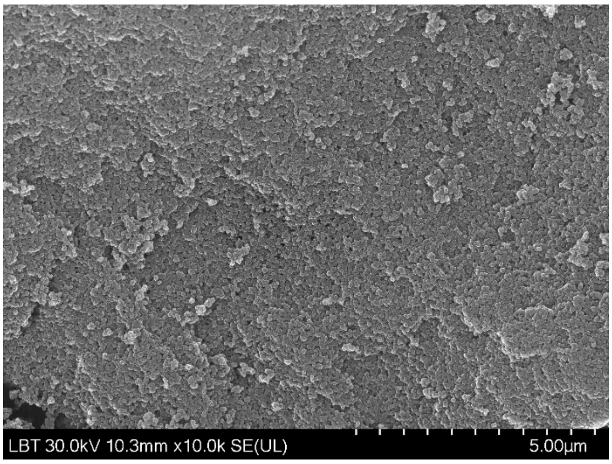

M.

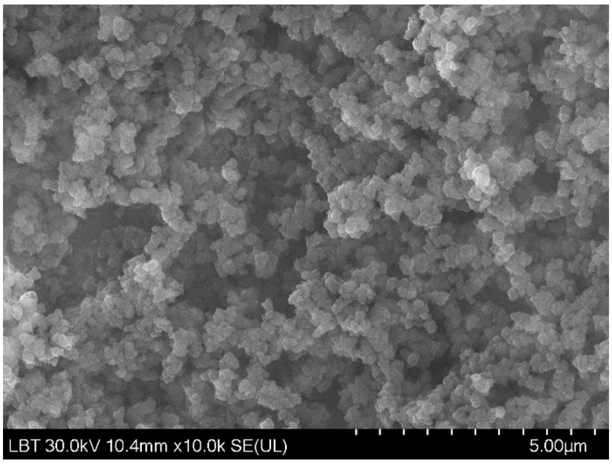

N.

Figure S1. Typical SEM images of the obtained polymer particles: A,B – MIP_M1; C,D – NIP_M1; E,F – MIP_M2; G,H – NIP_M2; I,J – MIP_S1; K,L – MIP_S2; M. – NIP_S1 and N. – NIP_S2.

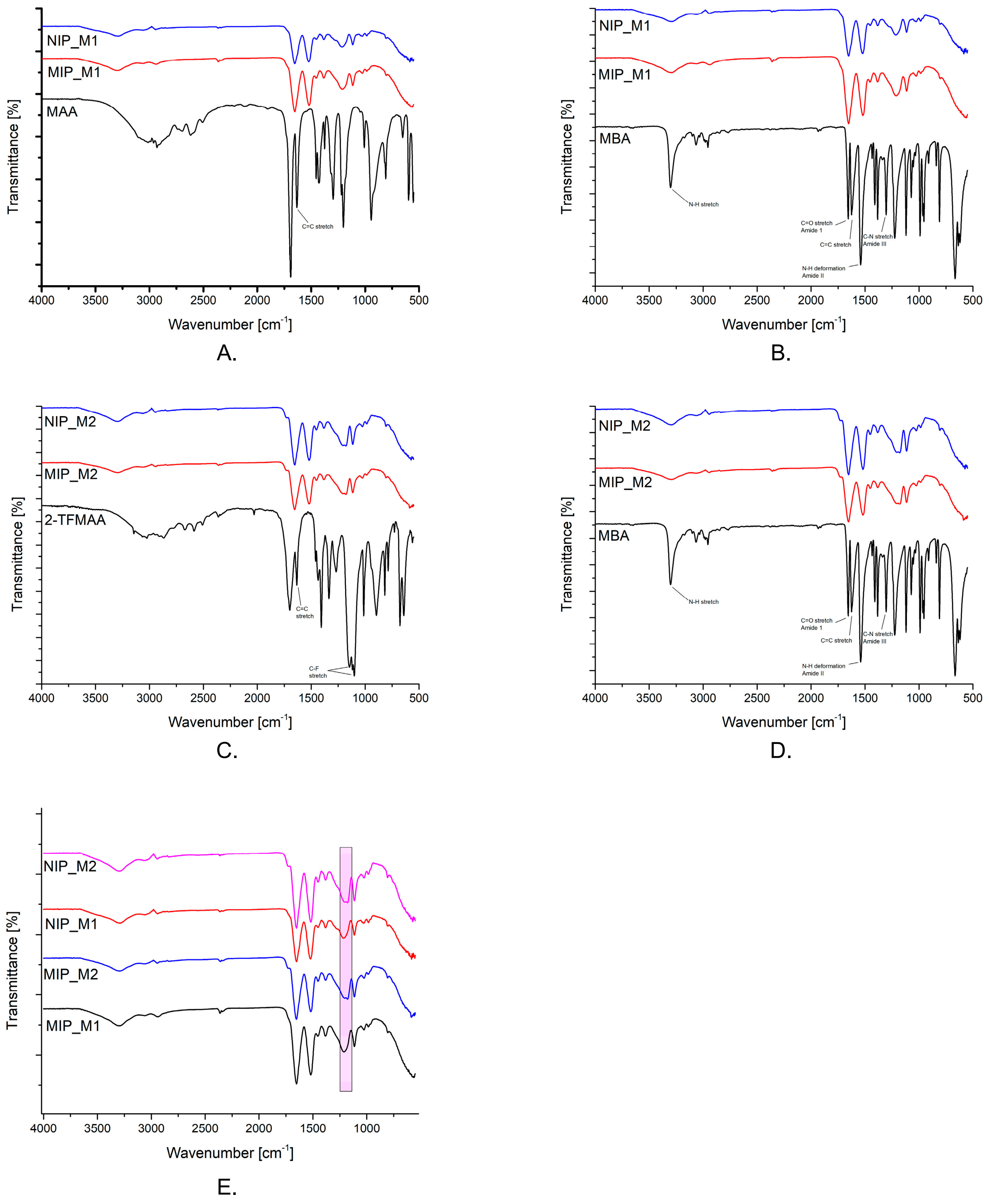

Figure S2. ATR-FT-IR spectra of synthesized polymers and key reaction components: A,B,E – MIP_M1 and NIP_M1; C,D,E – MIP_M2 and NIP_M2.

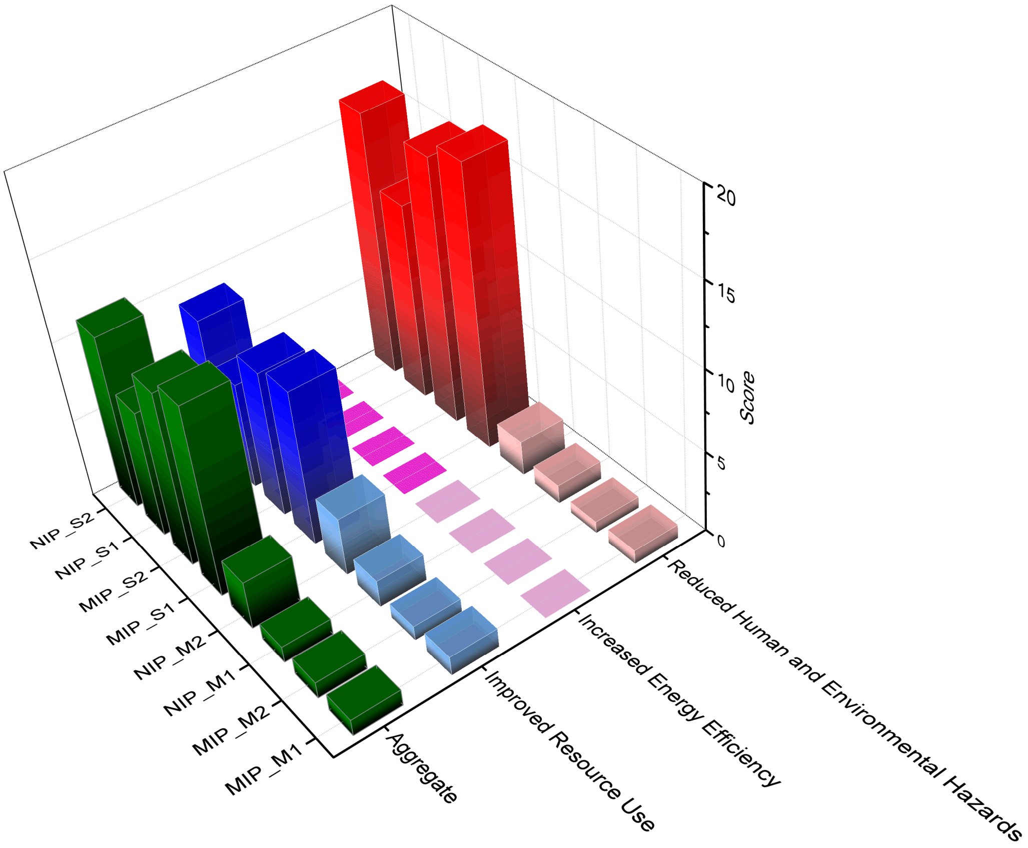

Figure S3. Comparisons of greenness for the solid state versus solution-based batch synthesis of imprinted and non-imprinted polymers.

Table S2. Comparisons of adsorption capacities and imprinting factors of the synthetized polymers using the mechanochemical and conventional approaches.

	Q (mg/g)[a]							
Polymer<br>β-blocker	**MIP_M1**	**NIP_M1**	**MIP_S1**	**NIP_S1**	**MIP_M2**	**NIP_M2**	**MIP_S2**	**NIP_S2**
ATNL (template)	18.59	1.89	19.05	15.92	19.11	6.39	14.76	15.68
PRNL	1.37	0.10	4.84	2.30	10.22	4.86	1.25	2.15
ALPRNL	1.69	0.78	4.16	0.43	9.74	4.29	1.60	3.41
CVDL	2.08	1.30	16.94	12.27	14.53	4.15	15.34	15.68
IF	**9.84**		**1.20**		**2.99**		**0.94**	

[a] Q – the adsorption capacities (mg/g) of the synthetized MIPs and NIPs.

Table S3. Physico-chemical and molecular properties of the studied β-blocker analogues and functional monomers predicted in MarvinSketch 5.1.3, ChemAxon Ltd.

	Log P	Polar surface area	Van der Waals surface area [Å^2]	Solvent accessible surface area [Å^2]	H-bond Donor/ Acceptor	Chemical structure
**ATNL**	0.43	84.58	436.67	541.09	3/4	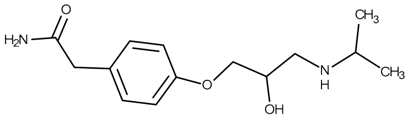
**PRNL**	2.58	41.49	425.72	526.99	2/3	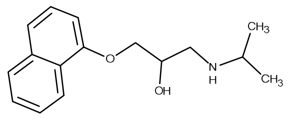
**ALPRNL**	2.69	41.49	426.67	517.96	2/3	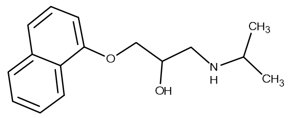
**CVDL**	3.42	75.74	604.24	737.14	3/5	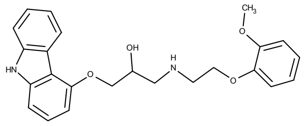
**MAA**	0.93	37.3	128.63	224.31	1/2	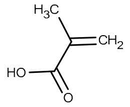
**2-TFMAA**	1.29	37.3	145.44	238.09	1/5	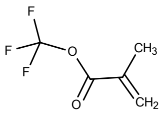
